# Progesterone and Mental Rotation Task: Is There Any Effect?

**DOI:** 10.1155/2014/741758

**Published:** 2014-04-10

**Authors:** Donatas Noreika, Inga Griškova-Bulanova, Aidas Alaburda, Mindaugas Baranauskas, Ramunė Grikšienė

**Affiliations:** ^1^Department of Neurobiology and Biophysics, Faculty of Natural Sciences, Vilnius University, 21 Čiurlionio Street, 03101 Vilnius, Lithuania; ^2^Department of General Psychology, Faculty of Philosophy, Vilnius University, 9/1 Universiteto Street, 01513 Vilnius, Lithuania

## Abstract

Mental rotation task (MRT) incorporates elements of spatial abilities, important in many professions, with people of both genders involved. Importantly, these are the areas where spatial tasks might be performed for long time periods; thus adverse effects of mental fatigue are highly unwanted. Substantial variation of MRT performance in relation to estrogen levels has been observed in many studies, whereas the role of progesterone remains elusive. Here we aimed to elucidate the effect of progesterone level on the long-duration (1.5 hours) performance of MRT. We included three groups of subjects: a group of males as a control, a group of females in their follicular phase (low progesterone) and a group of females in their luteal phase (high progesterone), MRT accuracy and response time, subjective fatigue ratings and cardiovascular measures together with 17**β**-estradiol and progesterone concentrations were analyzed. We found that subjective ratings of fatigue increased, performance accuracy increased, and mean response times decreased during the task in all groups. Females in luteal phase were significantly slower not only than men, but also than females in their follicular phase. An increase in subjective fatigue ratings was positively related to progesterone level—at higher progesterone levels, females felt more tired.

## 1. Introduction

Mental rotation task (MRT), originally described by Shepard and Metzler in 1971 [1], is one of the most studied visuospatial tasks, widely used in cognitive neuropsychology [2]. On average, men are more accurate and faster, compared to women in three-dimensional MRT [3–6]. This difference appears at an early age [7, 8] and persists through the lifespan [9, 10]. Mental rotation abilities can help predict success in such advanced field of performance as careers requiring highly developed spatial abilities or navigation in real and virtual environments (such as pilots, engineers, and architects) [11–14]. Importantly, these are the areas where representatives of both genders are involved and spatial tasks might be performed for long time periods; thus, adverse effects of mental fatigue are highly unwanted. Alternatively, when MRT is used in neuroimaging studies, the sufficient numbers of correct clean-data trials are necessary for averaging. This can be achieved through administration of long-duration experimental sessions, where mental fatigue can occur [15, 16].

Substantial variation of MRT performance is related to the phase of the menstrual cycle in females [17, 18]. The variation during the menstrual cycle seems to be mediated by 17*β*-estradiol levels [3, 17, 19–22], whereas the role of progesterone, if any, remains elusive. In previous studies, progesterone concentrations often covaried with 17*β*-estradiol as a consequence of the research designs that were used; yet progesterone failed to predict spatial performance in naturally cycling women [19, 23]. However, it was demonstrated that performance of MRT in hormonal contraceptive users depends on the specific progestins in contraceptive pills [24, 25].

Some evidence suggests that fatigue effects in females are modulated by progesterone. Murphy et al. [26] found elevated levels of progesterone in women with chronic fatigue syndrome. Freeman et al. [27], orally introducing progesterone at high doses, observed increased subjective fatigue. Söderpalm et al. [28] found increased feelings of fatigue after intramuscular injections of progesterone. On the other hand, Ziomkiewicz et al. [29] found higher subjective fatigue to be associated with lower, not higher, levels of progesterone during luteal phase of women menstrual cycle.

In this study we aimed to elucidate the effect of progesterone level on the long-term performance of MRT, hypothesizing that progesterone could have effects on behavioral MRT during task performance [30, 31]. For this reason, we included three groups of subjects: a group of males as a control, a group of females in their follicular phase (low progesterone), and a group of females in their luteal phase (high progesterone).

We collected data at three different levels, behavioral, subjective, and physiological. Behavioral measures, such as accuracy and response time, are the main parameters used to evaluate MRT performance [32, 33], as well as mental fatigue [34–36]. Assessment of subjective fatigue was implemented, as some studies found that progesterone level is associated with subjective fatigue ratings [28, 37]. Heart rate variability (HRV), as the physiological response related to activation of autonomic nervous system and being commonly used when measuring mental workload and mental effort assessment [38–40], which is directly associated with fatigue [41–44], was used to evaluate the physiological effect of prolonged performance of MRT.

## 2. Methods

### 2.1. Participants

49 subjects (18 men and 31 women) between 20 and 22 years (20.73 ± 0.7) took part in the study, all with normal or corrected to normal vision. For female participants, only healthy, nonpregnant, not using hormonal contraceptives, and experiencing regular menstrual cycle (mean duration 28.59, SD 2.13) women were included. Female participants were randomly assigned to take part in the experiment during one of the phases of their menstrual cycle: (1) follicular (FO; 15 participants); (2) luteal (LU; 16 participants). The time window for each phase was determined individually on the basis of the duration of subjects previous three-month cycle. It is known that the second part of menstrual cycle (from the ovulation to the onset of next menses) does not show high intersubject variability and lasts about 14 days [45]. Thus, the preliminary date of ovulation was determined (cycle duration minus 14 days) and, according to this, menstrual cycle was divided into two parts, follicular and luteal. The first half of the follicular part was used to avoid very high concentrations of 17*β*-estradiol and to have low progesterone levels. Six to eight days after the predicted ovulation, that is, the middle part of the luteal phase, was used expecting to capture the elevated progesterone concentration. The levels of salivary 17*β*-estradiol and progesterone were measured to validate phases retrospectively.

All the participants filled in the questionnaire, to exclude the possibility of chronic fatigue, substance abuse, chronic deficit of sleep, endocrinal illness, vision or hearing disorders, general health problems, and irregular menstrual cycle or hormonal contraceptives usage for women.

The study was approved by the Lithuanian Bioethics Committee. All the participants gave written informed consent prior to their participation. As all the subjects were Lithuanian speaking, all instructions for tests were presented in Lithuanian.

### 2.2. Experimental Task and Stimuli

**Figure 1:**
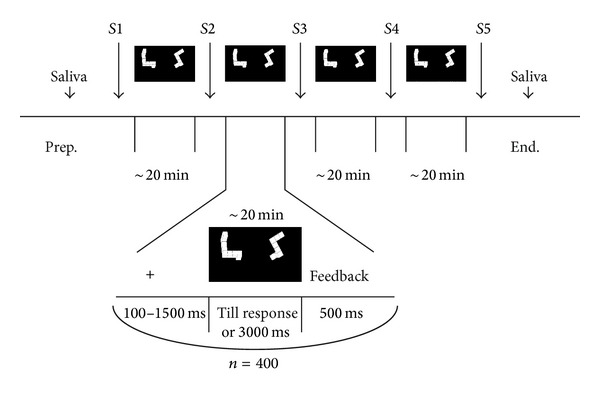
The scheme of the experiment;* S* denotes time points of subjective ratings.

Shepard and Metzler paradigm [1] was used for the investigation of mental rotation performance. Pairs of figures, rotated 90°, 135°, or 180° to each other, were presented to the subject. In half of the pairs the form of the figures was identical (but the figures were rotated to each other). In the other half, one figure was the mirror image of the other. The stimuli were composed of white cubes in black background and were taken from the “Library of Shepard and Metzler Type Mental Rotation Stimuli” [32]. The participants were instructed to press one of two buttons, indicating if the two figures are identical or different. The participants performed four blocks of the task, each containing 400 pairs of figures (and lasting ~20 min.). The pairs of figures inside a block were presented in random order, but the set of figures in all blocks was the same. Every trial started with a fixation cross, displayed for 100–1500 ms (randomly). Then a random pair of figures was presented, until the subject gave a response or till 3 s without a response passed. A feedback message, lasting 0.5 s (“Right!,” “Wrong,” or “The time for the response has expired!”) followed each response or 3 s without a response. The scheme of the experiment is depicted in Figure 1. E-Prime 2.0 software and PST Serial Response Box (Psychology Software Tools (PST), Inc.) were used for stimuli presentation and behavioral data collection. The outcome measures were accuracy (ACC, percent of correct answers) and response time of correct answers (RT). The progress of performance during the experiment was calculated by subtracting ACC and RT averages in the fourth block from ACC and RT averages in the first block.

### 2.3. Subjective Ratings

Between the task blocks the subjects were asked to rate four aspects of their subjective fatigue, by means of a visual analogue scale (VAS). Responding to a question “How tired are you feeling?” the subjects had to place a vertical mark on a horizontal line, anchored by maximum and minimum state descriptors (“No fatigue at all” and “Maximum fatigue”) at the ends. The scales were presented 5 times during the experiment (designated as* S*1 through* S*5 in the text; see Figure 1): (1) before the first MRT block, (2) before the 2nd block, (3) before the 3rd block, (4) before the 4th block, and (5) after the 4th block.

The progress of fatigue during the entire MRT load was calculated by subtracting values of* S*5 from* S*1.

### 2.4. Heart Rate Variability

The electrocardiogram (ECG) was monitored with PowerLab 3/80 polygraph (ADInstruments). Three disposable Ag—AgCl electrodes were placed according to the third derivation. The ECG signal was digitized at 1000 Hz, filtered with a 0.05–35 Hz bandpass filter, and inspected offline using LabChart 7.3 software (ADInstruments). Eight 5 min length records were selected for MR task analysis (one from beginning and one from the end of each of the four MR blocks). Time domain metrics included heart rate (HR), standard deviation of the RRIs (SDNN), and the square root of the mean squared successive heart period differences (RMSSD). Frequency domain measures included total power, high frequency (HF) component (0.15 Hz ≤ HF < 0.4 Hz), low frequency (LF) component (0.04 Hz ≤ LF < 0.15 Hz), normalized LF (LFnorm = LF/(LF + HF)), normalized HF (HFnorm = HF/(LF + HF)), and LF/HF ratio. The time and frequency domain measures of HRV were obtained using Kubios HRV 2.0 software (University of Kuopio, Finland). HRV spectrum was calculated with fast Fourier transform (FFT) based Welch's periodogram method.

### 2.5. Hormones Analysis

Salivary sex steroid (17*β*-estradiol and progesterone) levels were assayed to quantify free hormone levels, to validate self-reported cycle phases, and to compare hormone levels between groups. Salivary sampling is a noninvasive, simple, stress-free procedure approved as a useful method for the assessment of ovarian function [46–48]. The samples of saliva for the determination of free sex steroids were collected from the subjects at two time points in the study: at the beginning of the experiment and after the end of the main task. No saliva stimulants were used. Participants were asked not to eat, drink, chew gum, or brush their teeth for 30 min before sampling, but to rinse their mouth with cold water 5 min prior to sample collection. To avoid blood contamination, samples were not collected when oral disease, inflammation, or lesions were present. A minimum of 1 mL of saliva was collected into special tubes (IBL SaliCap). Tubes were stored at −24°C until assayed. Two saliva samples of each subject were mixed before the analysis to minimize possible effect of ultradian fluctuations of hormones concentration. The concentrations of free 17*β*-estradiol and free progesterone in saliva were determined by enzyme immunoassay for in vitro diagnostic quantitative determination in human saliva (IBL-International). The analytical sensitivity of the 17*β*-estradiol assay was 0.4 pg/mL and of the progesterone assay was 3.8 pg/mL. All samples were duplicated in the same assay.

### 2.6. Procedure

To minimize the effects of diurnal variations, all experimental sessions were performed in the afternoon, starting at 14.00–15.00 h. Participants were instructed to have an adequate sleep the night before the experiment and not to consume tonic or energy drinks two hours before the experiment. Participants filled a questionnaire to convey their health and emotional condition that day, time span since the last meal and its satiety (scales 1–5), and time span since the last coffee before the experiment.

In the preliminary session, several days before the main session, the subjects signed the informed consent, performed a training session, consisting of 96 pairs of stimulus figures (approximately 6 min), and filled the questionnaire, controlling for inclusion criteria.

After arriving at the main experimental session, participants were seated in an armchair in a soundproof, light-isolated chamber kept at a constant temperature (20–22°C), 80–83 cm from the computer monitor, and preparatory procedures, lasting 30–50 min, were started. Preparation included saliva sampling, a questionnaire about participant's physical and emotional state, and an application of electrodes (besides ECG, EEG was registered, not included in this analysis). MRT followed. The subjects performed 4 blocks of MRT (approximately 1.5 h).

Before, between, and after the task blocks the subjects were asked to rate their subjective fatigue. They had 1 minute to do it.

ECG was registered continuously during all experiment.

### 2.7. Statistical Analysis

The statistical analysis was performed with the STATISTICA 8.0 software (StatSoft, Inc., USA). ANOVA was used for evaluating effects of group and time-on-task. Sphericity assumption for repeated measures was checked with the Mauchly sphericity test and a correction for sphericity was applied (Greenhouse-Geisser adjustment) when necessary. Effect sizes were evaluated by partial eta squared (*η* ^2^). Post hoc Tukey tests were used when appropriate. Numbers, denoting means, are presented as mean ± standard error in the text. Pearson correlation analysis was performed to elucidate the relationship of hormonal levels with behavioral, subjective, and physiological measures.

## 3. Results

Men and women in both groups did not differ in their age (*P* > 0.1), body mass index (*P* > 0.1, min 17.7, max 27.8), time span since the last coffee (*P* > 0.1), time span since the last meal (*P* = 0.99), and subjectively evaluated last meal satiety (*P* > 0.1) (Table 1). Women did not differ in the duration of their menstrual cycle (*P* > 0.3) (Table 1). The hormonal profiles of female subjects corresponded to predefined phases of menstrual cycle: progesterone level was significantly higher in LU as compared to FO (*t* = 3.93, *P* < 0.001); 17*β*-estradiol level did not differ between groups (*t* = 0.53,   *P* = 0.60) (Table 1).

### 3.1. Behavioral Measures

Participants performed 4 blocks of MRT with 400 pairs of figures in each. The results, expressed as ACC (percent of correct responses) and RT, were subjected to 4 × 3 ANOVA (time-on-task: 4 blocks; group: men versus FO versus LU).

**Figure 2:**
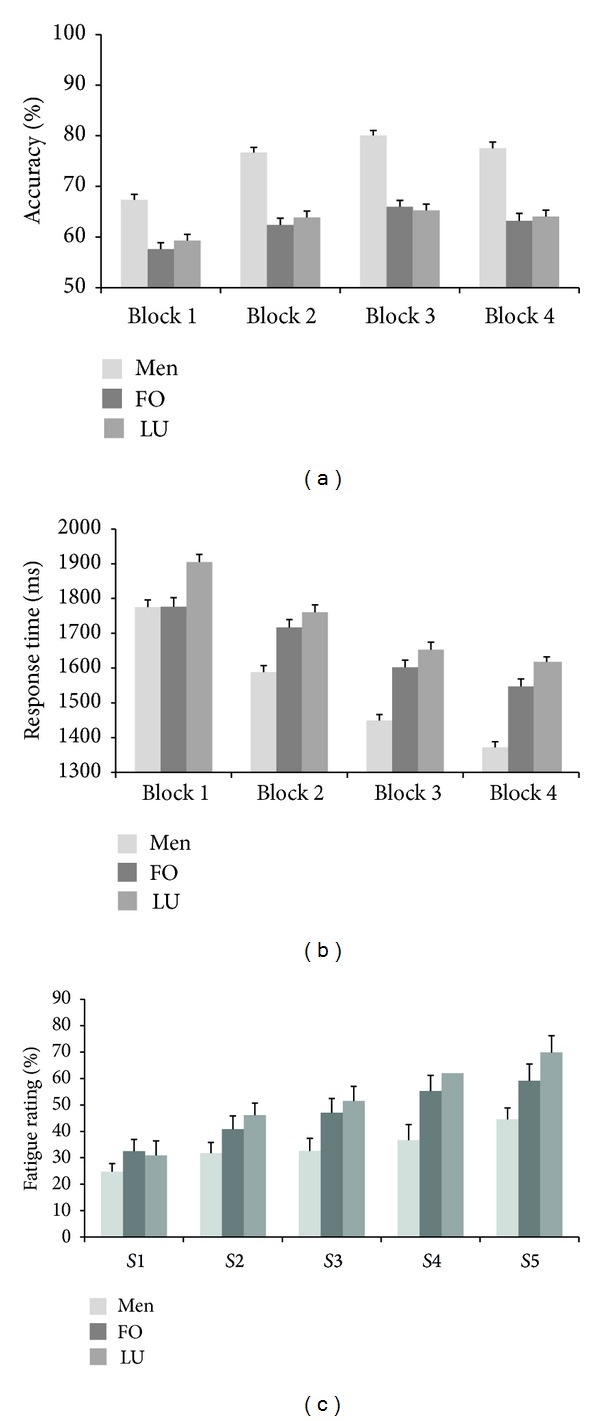
Mean values of mental rotation results ((a) accuracy, (b) mean response times) and mean values of subjective fatigue ratings (c) for men, follicular phase female (FO), and luteal phase female (LU) participants. Each block of the task contained 400 pairs of stimuli.* S*1–*S*5: subjective fatigue ratings before, between, and after MRT blocs. Vertical bars denote standard errors.

For accuracy, main effects of time-on-task (*F*(3, 1428) = 63.98, *P* < 0.001, *η* ^2^ = 0.12) and group (*F*(2, 476) = 55.64, *P* < 0.001, *η* ^2^ = 0.19), as well as the interaction of time-on-task and group (*F*(6, 1428) = 3.6, *P* < 0.01, *η* ^2^ = 0.01), were significant (Figure 2(a)). With increasing time-on-task, accuracy in all groups grew from the first to the third block and then decreased in the fourth block (Figure 2(a)). The overall accuracy progress during the task in men was the largest among the three groups: 10.2% from the first to the fourth block, as compared to 4.8% in FO and 4.6% in LU (pairwise progress comparison: *P* < 0.05 for men versus LU; *P* = n.s. for men versus FO and FO versus LU). Post hoc comparison between the groups revealed that men in accuracy (75.4 ± 1.0%) (all blocks) outperformed FO (62.3 ± 1.0%, *P* < 0.001) and LU (63.1 ± 1.0%, *P* < 0.001), with no significant difference between the two groups of women. Post hoc analysis in separate groups revealed that in all groups accuracy significantly increased from the first to the second block of MRT, followed by an insignificant increase from the second to the third block and an insignificant decrease from the third to the fourth block.

For mean response times, main effects of time-on-task (*F*(3, 1428) = 430.63, *P* < 0.001, *η* ^2^ = 0.47), group (*F*(2, 476) = 29.24, *P* < 0.001, *η* ^2^ = 0.11), and the interaction of time-on-task and group factors (*F*(6, 1428) = 12.83, *P* < 0.001, *η* ^2^ = 0.05) were significant. With increasing time-on-task, mean response times in all groups decreased from the first to the fourth block (Figure 2(b)). The overall progress of the mean response times of men was the highest among the three groups (−408 ms from the first to the fourth block, compared to −213 ms in FO and −290 ms in LU); *P* < 0.05 for men versus LU; *P* = n.s. for men versus FO and FO versus LU. Post hoc comparison between the groups revealed that LU (1734 ± 18 ms) were slower than FO (1660 ± 18 ms, *P* = 0.01) and men (1546 ± 17 ms, *P* < 0.001); FO were slower than men (*P* < 0.001). Post hoc analysis of separate groups revealed that decrease of the response time in consecutive blocks was significant in all groups except LU, where the decrease from the third and fourth task blocks was not significant (*P* = 0.40).

Correlation analysis between hormones' levels and behavioral results in females revealed that slower mean response times corresponded to higher 17*β*-estradiol levels (*r* = 0.42, *P* = 0.04) and there was a tendency for longer RTs to be observed with higher progesterone levels (*r* = 0.37, *P* = 0.07). There were no significant relationships between 17*β*-estradiol (*r* = 0.30, *P* = 0.15) and progesterone (*r* = −0.10, *P* = 0.65) levels with mean accuracy.

### 3.2. Subjective Ratings

Participants rated their subjective fatigue on a scale five times during the experiment. The results, expressed as percent of the VAS, were subjected to 5 × 3 ANOVA (time-on-task: 5 measurements; group: men versus FO versus LU).

The effects of time-on-task (*F*(4, 180) = 57.40, *P* < 0.001, *η* ^2^ = 0.56), group (*F*(2, 45) = 3.42, *P* = 0.04, *η* ^2^ = 0.13), and the interaction of time-on-task with group were significant (*F*(8, 180) = 2.66, *P* < 0.01, *η* ^2^ = 0.11). Mean values of subjective fatigue ratings are presented in Figure 2(c). The overall progress (scale 5 minus scale 1; see methods) of subjective fatigue in LU group (39.0%) was significantly larger as compared to men (21.2%, *P* < 0.05) but did not differ significantly between LU and FO (26.7%). Post hoc analysis revealed that LU (52.1 ± 5.0%) rated their subjective fatigue significantly higher than men (34.0 ± 4.9%, *P* = 0.04), with insignificant difference between the other two pairs of groups (LU versus FO (46.9 ± 5.2%) and men versus FO).

Correlation analysis indicated that higher progesterone concentration was significantly associated with higher change in subjective fatigue ratings (*r* = 0.45, *P* = 0.02); that is, the progress of subjective fatigue increased with increasing progesterone. No other significant relationships between hormones and subjective fatigue ratings were found.

### 3.3. Heart Rate Variability Measures

Eight 5 min length records were used for ECG analysis (one from the beginning and one from the end of each of the four MRT blocks). The results, expressed as heart rate, heart rate variability (as SDNN and RMSSD), total spectral power, low frequency power, high frequency power, normalized LF, normalized HF, and LF/HF ratio, were subjected to 8 × 3 ANOVA (time-on-task: 8 samples; group: men versus FO versus LU). The time-on-task analysis revealed that, with increasing time-on-task, HR and HFnorm decreased and SDNN, RMSSD, total power, LF, HF, LFnorm, and LF/HF ratio increased (Table 2; see Supplement A in the Supplementary Material available online at http://dx.doi.org/10.1155/2014/741758).

The group factor was not significant for any of the parameters (Table 2). However LU tended to have higher heart rate and lower heart rate variability (SDNN, RMSSD, total power, and LF) values (for details see Supplement A).

There were no significant relationships between hormones and mean values of cardiovascular parameters or cardiovascular parameters' changes during the task.

## 4. Discussion

In this study we evaluated the effect of progesterone level on the long-term performance of MRT. We included three groups of subjects: a group of males as a control with high-performance level, a group of females in their follicular phase (low progesterone), and a group of females in their luteal phase (progesterone levels on average were more than three times higher than in follicular phase). In contrast to earlier MRT studies [33, 49–51] we used long-duration (1600 pairs of figures and ~1,5 h of performance) MRT task.

We found that performance accuracy increased and mean response times decreased in all groups during the task. This indicates that MRT practice effect [33, 49–51] occurred even during single prolonged MRT session.

On the behavioral level, men showed higher accuracy and shorter response times than either of the female groups, confirming findings from earlier studies that men outperform women in MRT on average [52–54]. Substantial variation of MRT performance in females was previously related to the phase of the menstrual cycle [17, 18]; several studies demonstrated negative MRT performance relationship with estrogens but not progesterone in naturally cycling women [3, 55]. However, in our recent study we demonstrated that performance of MRT in hormonal contraceptive users depends on the characteristics of synthetic progestins in contraceptive pills [24]. This suggested that significant effect of progesterone or progesterone metabolites on MRT performance could be expected.

The level of 17*β*-estradiol was similar in both female groups in our current study, as it was expected [3, 55]. FO and LU did not differ in MRT accuracy; nevertheless, higher 17*β*-estradiol levels corresponded to longer response times. However, females in LU were significantly slower not only than men, but also than females in FO (Figure 2(b)) corresponding to our previous observations in females on progestin's containing contraceptives [24]. This is also in line with study of Freeman et al. [37], where a high dose of progesterone (1200 mg) impaired psychomotor performance and increased general fatigue. In addition, slower performance in LU and 17*β*-estradiol and progesterone correlation with response time partly agree with results of Simić and Santini [18]. These authors found delayed task performance times during preovulation and midluteal phase (theoretically, high estrogen and progesterone phases); unfortunately the concentrations of estrogens and progesterone were not measured in their study. It should be noted that, in the studies where the effects of menstrual cycle phase or levels of sex steroids on MRT performance were investigated, accuracy parameter predominated over the response time [55–57]. However, as an increase in response time with increasing angular difference is a sign of mental rotation process per se [1], this measure cannot be ignored when evaluating effect of various factors on MRT. Our results point to the fact that higher level of progesterone in a sample of naturally cycling young females might contribute to slower MRT performance. To note, in the present study LU differed in mean response times from males and FO already during the first block (Figure 2(b)). This indicates the importance of progesterone level (together with 17*β*-estradiol level) for MRT task evaluation even when short-duration trials are being used.

Men started the task with higher accuracy and shorter response times and their progress was also the highest between the three groups (for accuracy, 10.2% from the first to the fourth block, compared to 4.8% for FO and 4.6% for LU; for response time, 21.5% from the first to the fourth block, compared to 12.1% for FO and 15.2% for LU). No difference in progress between FO and LU was observed (Figures 2(a) and 2(b)). However, in contrast to previous studies demonstrating that the relationship between hormones and MRT performance disappeared with repeated testing (e.g., [3, 58]) the differences between men and women, as well as the slowest RTs in LU group, remained during the course of the whole prolonged task in our study.

The subjective fatigue gradually increased in all groups during the MRT task. The highest values of subjectively rated fatigue and the highest increase of fatigue ratings during the task were observed in LU group, although no statistically significant differences were obtained between female groups. However, the increase in subjective fatigue ratings was positively related to progesterone level—at higher progesterone levels, females felt more tired. The comparable observation was made by Freeman et al. [37]: in their study plasma levels of progesterone metabolite allopregnanolone were significantly correlated with measures of fatigue. Several studies showed that in the luteal phase progesterone and its metabolite allopregnanolone positively correlated with fatigue [28, 30, 31] and negatively correlated with arousal [31].

We used cardiovascular measures to evaluate an activation of autonomic nervous system during the prolonged MRT task. Statistical analysis of these measures revealed significant effect of time-on-task but no effect of group. This indicates that autonomic nervous system in all groups adapted to the MRT task in a similar way. Different ECG parameters are considered to grope different aspects of the physiological state—HR, SDNN, RMSSD, and HF seemed to be more vulnerable to stress (tension) levels and LF and LF/HF ratio more to the mental effort, needed for the task. Heart rate tended to decrease and heart rate variability (measured as SDNN and RMSSD) along with HF tended to increase during the task. This might be explained as an adaptation to the task and diminished stress level during the task [59–62]. LF, LFnorm, and LF/HF ratio are thought to be influenced by both parasympathetic and sympathetic branches of ANS [60, 61]. However, some researchers [63, 64] found mental fatigue to increase sympathetic activity and in this case LF increase with time-on-task might indicate increasingly larger effort (because of fatigue) to complete the task during MRT.

Interestingly, LU tended to have higher HR and lower HRV values (SDNN, RMSSD, total power, and LF); this tendency is compatible with higher general fatigue levels in the luteal phase, but additional investigation is needed to resolve this issue. It is difficult to compare the observed trend with earlier studies, as estimation of HRV components during the menstrual cycle was performed during the resting state before but not during the task performance [65, 66].

To sum up, our results suggest that high progesterone during luteal phase in young healthy women has effect on the MRT performance by slowing the responses. This should be taken into account in further studies.

## Supplementary Material

The dynamics of heart rate and heart rate variability during the task in three experimental groups.

## Figures and Tables

**Table 1:** Demographic characteristics of the participants.

	Men	FO	LU
Age	20.8 ± 0.8	20.6 ± 0.2	20.8 ± 0.1
Body mass index, kg/m^2^	22.8 ± 0.9	21.0 ± 0.7	20.4 ± 0.8
Duration of the menstrual cycle, days	—	29.2 ± 0.5	28.30 ± 0.60
Progesterone, pg/mL	—	67.8 ± 5.2	220.6 ± 40.1
17*β*-Estradiol, pg/mL	—	5.23 ± 0.55	4.87 ± 0.37
Time span since last coffee, h	5.44 ± 0.48	4.73 ± 0.50	4.75 ± 0.54
Time span since last meal, h	3.44 ± 1.05	3.32 ± 0.63	3.25 ± 0.30
Satiety of the last meal, scales 1–5	3.77 ± 0.24	3.27 ± 0.27	3.25 ± 0.30

**Table 2:** The influence of time-on-task (8 samples) and group (men, FO, and LU) on heart rate and heart rate variability. Significant differences are in bold.

HRV parameter		Time-on-task			Group		
	*F*	*P*	*η* ^2^	Direction	*F*	*P*	*η* ^2^
HR, beats/min	21.96	**<0.001**	0.37	↓	0.43	0.65	0.02
SDNN, msSDNN, ms	11.25	**<0.001**	0.23	↑	1.86	0.17	0.09
RMSSD, ms	7.06	**<0.001**	0.16	↑	1.49	0.24	0.07
Total power, ms^2^	6.39	**<0.001**	0.15	↑	1.70	0.20	0.08
LF, ms^2^	6.16	**<0.001 **	0.14	↑	1.81	0.18	0.09
HF, ms^2^	3.38	**0.002**	0.08	↑	0.54	0.58	0.03
LFnorm, n.u.	3.78	**<0.001 **	0.09	↑	0.24	0.79	0.01
HFnorm, n.u.	3.66	**<0.001 **	0.09	↓	0.24	0.79	0.01
LF/HF ratio	3.41	**0.010**	0.09	↑	1.14	0.33	0.06

HR: heart rate, SDNN: standard deviation of the RRIs, RMSSD: the square root of the mean squared successive heart period differences, HF: high frequency component, LF: low frequency component, LFnorm: normalized LF, and HFnorm: normalized HF.
