# Supplementary figures and images for: Progesterone and Mental Rotation Task: Is There Any Effect?
Source: Biomed Res Int. 2014 Apr 10;2014:741758. doi: 10.1155/2014/741758 (PMC4004113; doi:10.1155/2014/741758)

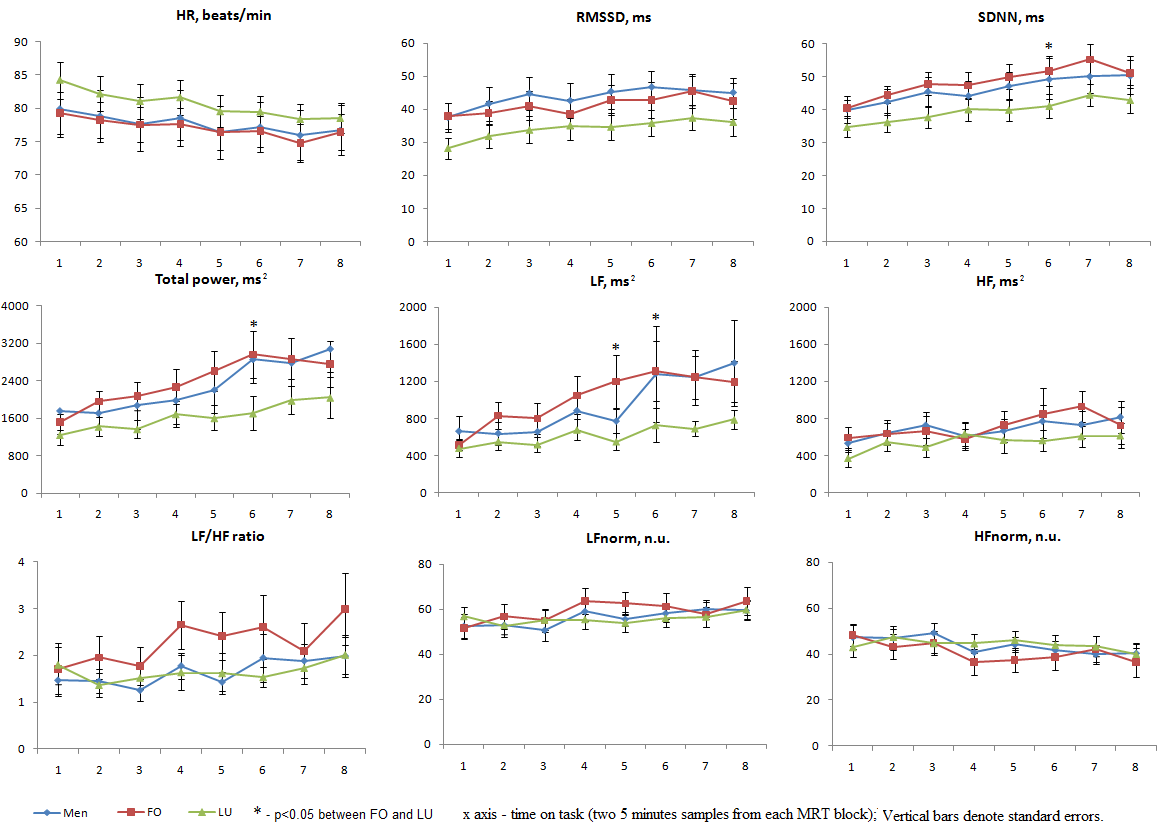

Supplement: Supplementary file 1 — The dynamics of heart rate and heart rate variability during the task in three experimental groups. [file 741758.f1.docx]
